# Treatment and prevention of malaria in pregnancy in the private health sector in Uganda: implications for patient safety

**DOI:** 10.1186/s12936-016-1245-2

**Published:** 2016-04-14

**Authors:** Anthony K. Mbonye, Esther Buregyeya, Elizeus Rutebemberwa, Siân E. Clarke, Sham Lal, Kristian S. Hansen, Pascal Magnussen, Philip LaRussa

**Affiliations:** Ministry of Health, Directorate of Clinical and Community Services, Kampala and Department of Community and Behavioural Sciences, School of Public Health, Makerere University, Box 7272, Kampala, Uganda; Department of Disease Control and Environmental Health, School of Public Health, Makerere University, Kampala, Uganda; Department of Health Policy, Planning and Management, School of Public Health, Makerere University, Kampala, Uganda; Department of Disease Control, London School of Hygiene and Tropical Medicine, Keppel Street, London, WC1E 7HT UK; Department of Global Health and Development, London School of Hygiene and Tropical Medicine, 15-17 Tavistock Place, London, WC1H 9SH UK; Institute for International Health, Immunology and Microbiology, Centre for Medical Parasitology and Institute for Veterinary Disease Biology, Faculty of Health and Medical Sciences, University of Copenhagen, Copenhagen, Denmark; College of Physicians and Surgeons, Columbia University, New York, USA

## Abstract

**Background:**

Malaria in pregnancy is a major public health problem in Uganda; and it is the leading cause of anaemia among pregnant women and low birth weight in infants. Previous studies have noted poor quality of care in the private sector. Thus there is need to explore ways of improving quality of care in the private sector that provides almost a half of health services in Uganda.

**Methods:**

A survey was conducted from August to October 2014 within 57 parishes in Mukono district, central Uganda. The selected parishes had a minimum of 200 households and at least one registered drug shop, pharmacy or private clinic. Data was collected using a structured questionnaire targeting one provider who was found on duty in each selected private health facility and consented to the study. The main variables were: provider characteristics, previous training received, type of drugs stocked, treatment and prevention practices for malaria among pregnant women. The main study outcome was the proportion of private health facilities who prescribe treatment of fever among pregnant women as recommended in the guidelines.

**Results:**

A total of 241 private health facilities were surveyed; 70.5 % were registered drug shops, 24.5 % private clinics and 5.0 % pharmacies. Treatment of fever among pregnant women in accordance with the national treatment guidelines was poor: 40.7 % in private clinics, decreasing to 28.2 % in drug shops and 16.7 % at pharmacies. Anti-malarial monotherapies sulphadoxine-pyrimethamine and quinine were commonly prescribed, often without consideration of gestational age. The majority of providers (>75 %) at all private facilities prescribed SP for intermittent preventive treatment but artemisinin-based combination therapy was prescribed: 8.3, 6.9 and 8.3 % respectively at drug shops, private clinics and pharmacies for prevention of malaria in pregnancy. Few facilities had malaria treatment guidelines; (44.1 % of private clinics, 17.9 % of drug shops, and 41.7 % at pharmacies. Knowledge of people at risk of malaria, *P* = 0.02 and availability of malaria treatment guidelines, *P* = 0.03 were the factors that most influenced correct treatment of fever in pregnancy.

**Conclusion:**

Treatment of fever during pregnancy was poor in this study setting. These data highlight the need to develop interventions to improve patient safety and quality of care for pregnant women in the private health sector in Uganda.

## Background

Malaria in pregnancy is a major public health problem in Uganda [1], responsible for anaemia among pregnant women and low birth weight in infants [2]. The current policy for treating malaria in pregnancy in the first trimester is to give quinine 10 m/kg; and during the second and third trimester, artemisinin-based combination therapy (ACT) is recommended. For malaria prevention in Uganda, the policy recommends at least three doses of sulfadoxine-pyrimethamine (SP) as intermittent preventive treatment (IPTp), and use of insecticide-treated bed nets (ITNs) [3]. Adherence to the guidelines (not to give ACT or any anti-malarial monotherapies during the first trimester) would ensure effective treatment of malaria and patient safety.

Although utilization of health-facility based interventions like antenatal care (ANC) and delivery care is generally poor [4], SP-IPTp has one of the lowest implementation rates compared to other interventions in pregnancy and childhood. Recent data shows that low utilization of essential health services leads to missed opportunity. For example, 47.6 % pregnant women attend the required four antenatal care visits; 26.7 % receive the two doses of SP-IPTp while 55.5 % get at least two doses of tetanus toxic vaccination [4]. Previous studies have assessed factors associated with low utilization of health-based interventions, including maternity services, and have documented socio-cultural factors, economic, constraints, negative perceptions, poor quality of care and long distances to health units [5–14].

Several studies have implemented private-sector interventions aimed at increasing access to malaria prevention services in Uganda. One study implemented at a community level, assessed whether drug shops, traditional birth attendants, community health workers and peer adolescent mobilizers would increases access to essential maternity services. The study demonstrated increased access to two doses of SP with an impact on pregnancy outcomes [15, 16]. It was further shown that the distribution of SP at a community level did not negatively affect access to essential health services at health facilities [2]. Another study assessed the feasibility of private midwives in prevention of mother-to-child transmission of human immune deficiency virus (HIV) [17]. The study showed that private midwives were already providing essential maternity services; and they were trusted by their communities. Despite this scenario, access to HIV and malaria preventive services was low among the poor, the young and the less educated women. Following this study, it was recommended that private midwives be given refresher courses, to improve the quality of services [17].

It has also been shown that many patients (including pregnant women) with febrile illnesses receive anti-malarial drugs and only 34 % of malaria patients received an appropriate ACT regimen. This practice is due to the continued practice of presumptive treatment, inadequate training on malaria management and lack of knowledge that artemether-lumefantrine (Coartem^®^) was the recommended first-line treatment for malaria in Uganda [18].

Based on the above evidence, and the need to scale up services to achieve universal coverage, Uganda formulated a public–private partnership policy and is currently discussing modalities for its implementation [1]. Studies to guide this process are required, including exploring the feasibility of involving the private health sector in prevention and control of malaria in pregnancy. The main objective of the study was to assess quality of care in the private sector for patients seeking care in this outlet [19]. Specifically the sub-study assessed existing malaria prevention and treatment practices in the private sector in order to design effective interventions to improve access and quality of care for pregnant women seeking treatment in this sector.

## Methods

### Study design and setting

The study design is reported elsewhere [19], but briefly the study was undertaken in Mukono district, bordering Lake Victoria in Central Uganda. The district area is endemic for malaria, with high disease prevalence around May–June and November–December. The district population is estimated at 565,700 with an annual growth rate of 2.3 % [16]. Most people, 88 %, live in the rural areas; access to ANC (at least one visit) is high, 94.1 %; however, only 26.7 % of pregnant women receive the two or more doses of SP as recommended by policy [4].

A survey was conducted from August to October 2014. A list of all parishes from which sampling was done was obtained from Uganda Bureau of Statistics, Kampala. The criteria for selecting study parishes were: (i) contained more than 200 households to ensure a sufficient number of patients visiting the facilities; (ii) contained a health centre II, the lowest public health facility where early treatment and referral is sought; and (iii) contained at least one registered drug shop, pharmacy or private clinic. In total, 57 parishes with 242 private facilities were selected to implement the study A structured questionnaire was used to collect data targeting one provider who was found on duty in each selected private health facility.

Both the facility and its health workers were assessed using a semi-structured questionnaire. One staff in each private facility (drug shops, private clinics and pharmacies) and higher level (Health Centre II, III, IV and hospitals) that consented to the study was interviewed. In higher referral facilities the study targeted in-charges or deputies in the higher level facilities and whoever was present on the day of the interview.

The study outcome was the proportion of private health facilities who prescribe treatment of fever among pregnant women as recommended in the guidelines (quinine, 10 m/kg in the first trimester; and ACT in the second and third trimester [3]).

### Data collection methods

Data was captured on the following variables: provider characteristics, previous training received, type of drugs stocked, knowledge on treatment and prevention of malaria in pregnancy. The interviews were conducted by experienced research assistants, who underwent refresher training for 5 days on research techniques, and study procedures; and participated in the pretesting and revision of the questionnaire tools before actual field work. Since all private providers had a working knowledge of English, the questionnaires were administered in English language. All aspects of data collection were supervised.

### Sample size calculation

Sample size calculation was based on the proportion of private health facilities prescribing treatment of fever among pregnant women as recommended in the guidelines (quinine, 10 m/kg in the first trimester; and ACT in the second and third trimester) estimated at 25 %. In order to estimate the proportion with a ±7 % absolute precision, at a power of 80 and 5 % level of significance (two-sided), a minimum of 232 private health facilities were required.

### Statistical methods

Data was entered using Microsoft Access 2007 (Microsoft Inc., Redmond, Washington) and analysed using STATA version 11.0 (STATA Corporation, College Station, Texas). Proportions on key variables were calculated using univariate analyses; factors associated with treatment of fever among pregnant women were assessed using bivariate analyses. Variables with a *p* value <0.05 were included in a logistic regression model that assessed factors associated with correct treatment of fever among pregnant women. The robustness of the regression model was tested using a Hosmer–Lemeshow goodness of fit Chi Square test and considered significant at a *P* >0.05.

### Ethics

Ethical approval for the research was granted from review boards at the Uganda National Council of Science and Technology (Ref: ss 3529). Written informed consent was sought prior to the interviews. The collected data could only be accessed by the researchers to ensure confidentiality.

## Results

### Characteristics of private health facilities

A total of 241 private health care facilities were surveyed; 70.5 % were drug shops; 24.5 % were private clinics and 5.0 % were pharmacies. The majority, >73 % were registered; and most (>75 %) were registered with the National Drug Authority; others were registered at the district level.

All the facilities sold anti-malarial drugs (Table 1). Almost all were stocked with artemisinin-based combinations (artemether-lumefantrine), the first-line anti-malarial drug and the majority, 82 % were stocked with quinine (the second-line anti-malarial drug). The majority, over 78 % also stocked SP. However, a sizeable proportion also stocked other anti-malarial monotherapies which are no longer recommended: such as chloroquine, 12.4 % at drug shops and amodiaquine (Camoquine^®^, 10.2 % at private clinics). Most of the facilities were busiest in the morning and evenings. The majority of private clinics (84.8 %) had patient registers; but only a few of the drug shops had a patient register, 21.2 %. Few of the private clinics, 25 % or drug shops, 16 % had stock cards. RDTs were available for malaria diagnosis in more than 75 % of pharmacies and private clinics, and in 43.8 % of registered drug shops. In contrast, only 44 % of private clinics, 42 % of pharmacies and 18 % of drug shops were equipped with the current national malaria treatment guidelines.

**Table 1:** Characteristics of private health care facilities

Characteristics	Registered drug shops N = 170	Private clinics N = 59	Pharmacy N = 12
Type of facility	170 (70.5 %)	59 (24.5)	12 (5.0 %)
Facility location	130 (76.5 %)	53 (89.8 %)	11 (91.7 %)
Urban rural	40 (23.5 %)	6 (10.2 %)	1 (8.3 %)
Facilities registered	125 (73.5 %)	54 (91.5 %)	12 (100 %)
Where facilities are registered			
District (receipt seen)	29 (23.6 %)	8 (14.9 %)	0 (0.0 %)
NDA (license seen)	93 (75.6 %)	45 (83.3 %)	12 (100 %)
No response	1 (0.9 %)	1 (1.9 %)	0 (0.0 %)
Busiest time at this facility			
Morning (up to 12 pm)	46 (27.1 %)	25 (42.4 %)	9 (75.0 %)
Afternoon (12–5 pm)	10 (5.9 %)	7 (11.9 %)	0 (0.0 %)
Evening (5–7 pm)	19 (46.5 %)	17 (28.8)	1 (8.3 %)
Night (7–12 am)	20 (11.8 %)	5 (8.5 %)	2 (16.7 %)
Facilities with a patient register	36 (21.2 %)	50 (84.8 %)	7 (58.3 %)
Facilities with stock control cards	27 (15.9)	15 (25.4)	7 (58.3)
Facilities with malaria treatment guidelines	30 (17.9)	26 (44.1)	5 (41.7)
Facilities with a thermometer	160 (94.1)	58 (98.3)	11 (91.7)
Facilities with an RDT for malaria diagnosis	74 (43.8)	54 (89.8)	9 (75.0)
Facilities selling anti-malarial drugs	170 (100)	59 (100)	7 (100)
Chloroquine	21 (12.4)	4 (6.8)	5 (41.7)
Fansidar (SP)	134 (78.8)	52 (88.1)	12 (100)
Camoquin	11 (6.5)	6 (10.2)	1 (8.3)
Quinine	141 (82.9)	53 (89.8)	11 (91.7)
ACT	166 (97.7)	57 (96.6)	12 (100)

### Provider characteristics

Providers were predominantly female, especially in drug shops, 87.7 % compared to 56.0 % in private clinics and 58.3 % in pharmacies (Table 2). Similarly, the majority of providers at drug shops 52.9 % had not progressed beyond secondary education while at private clinics and pharmacies, the majority had attained tertiary education. Most providers at drug shops were of lower professional cadre (nursing assistants, nursing aides, enrolled nurses/midwives); while at private clinics providers were of a higher professional cadre (registered nurses/midwives and clinical officers).

**Table 2:** Providers characteristics at private health care facilities

Providers characteristics	Registered drug shops N = 170	Private clinics N = 59	Pharmacy N = 12
Sex			
Male	21 (12.3)	26 (44.0)	5 (41.7)
Female	149 (87.7)	33 (56.0)	7 (58.3)
Education			
Secondary	90 (52.9)	12 (20.3)	0 (0.0)
Tertiary	80 (47.1)	43 (72.9 %)	10 (83.3)
University	0 (0.0)	4 (6.8)	2 (16.7 %)
Level of professional training			
Nursing aide	11 (6.5)	0 (0.0)	11 (4.6)
Nursing assistant	82 (48.2)	6 (10.2)	2 (16.7)
Enrolled nurse/midwife	52 (30.6)	6 (10.2)	2 (16.7)
Registered nurse/midwife	6 (3.5)	7 (11.9)	0 (0.0)
Clinical officer	4 (2.4)	14 (23.7)	2 (16.7)
Doctor	0 (0.0)	3 (5.1)	0 (0.0)

### Treatment and prevention of febrile illnesses in pregnancy

Private providers were asked what treatment they give to pregnant women who present with fever to their facilities. Treatment of pregnant women with fever according to the guidelines was 40.7 % at private clinics, 28.2 % at drug shops and 16.7 % at pharmacies. A big proportion, 45.9 % of drug shops prescribed SP as first-line treatment for malaria to pregnant women; while 2.9 % prescribed ACT. The majority of providers (>75 %) at all private facilities prescribed SP for intermittent preventive treatment (IPTp) but artemisinin-based combination therapy (ACT) was also prescribed: 8.3, 6.9 and 8.3 % respectively at drug shops, private clinics and pharmacies for prevention of malaria in pregnancy.

Knowledge on pregnant women being vulnerable to malaria was low, 11.2 % at drug shops, 6.8 % at private clinics and none at pharmacies. Most providers, >89 % recommended ITNs to pregnant women for malaria prevention (Table 3). The following factors were associated with correct treatment given to pregnant women with fever: the type of facility (drug shop), *P* = 0.04; knowledge of people vulnerable to malaria, *P* = 0.02; and availability of a malaria treatment guideline, *P* = 0.03 (Table 3).

**Table 3:** Level of knowledge, treatment and prevention of malaria in pregnancy at private health facilities

Knowledge, treatment and prevention of malaria in pregnancy	Registered drug shops N = 170	Private clinics N = 59	Pharmacy N = 12
Knowledge about of people at risk of malaria			
All people	24 (14.1)	10 (17.0)	9 (41.7)
Children (0–5)	58 (43.1)	30 (50.9)	5 (41.7)
Children (6–15)	61 (35.9)	10 (17.0)	1 (8.3)
Adults	8 (4.7)	4 (6.8)	0 (0.0)
Pregnant women	19 (11.2)	4 (6.8)	0 (0.0)
Treatment given to pregnant women with fever according to the guideline			
First trimester give quinine and second trimester give (ACT) as recommended by the guideline	48 (28.2)	24 (40.7)	2 (16.7)
Drugs given to pregnant women who present with fever			
ACT	5 (2.9)	4 (6.8)	1 (8.3)
SP	78 (45.9)	11 (18.6)	3 (25.0)
Quinine	12 (7.1)	7 (11.9)	2 (16.7)
Paracetamol	12 (7.1)	4 (6.8)	2 (16.7)
Other (amoxicillin, folic acid, iron)	15 (8.8)	9 (15.3)	2 (16.7)
Anti-malarial drug given to for malaria prevention			
Quinine	5 (3.0)	0 (0.0)	1 (8.3)
ACT	14 (8.3)	4 (6.9)	1 (8.3)
SP	128 (76.2)	52 (89.2)	9 (75.0)
Other (amoxicillin, chloroquine, vitamin B-complex)	21 (12.5)	2 (3.5)	1 (8.3)
Malaria prevention interventions providers recommend to pregnant women			
Insecticide treated nets (ITNs)	152 (91.0)	52 (89.7)	12 (100)
In-door residual spray (IRS)	2 (2.4)	2 (3.5)	0 (0.0)
Advice on good nutrition	3 (1.8)	2 (3.5)	0 (0.0)
Close windows early	3 (1.8)	1 (1.7)	0 (0.0)

Factors associated with treatment (according to guideline) given to pregnant women with fever	Statistical significance
Drug shop	*P* = 0.04
Private clinic	*P* = 0.10
Availability of malaria treatment guideline	*P* = 0.03
Presence of a functional laboratory	*P* = 0.90
Presence of a functional microscope	*P* = 0.80
Knowledge of people vulnerable to malaria	*P* = 0.02
Professional qualification of the provider	*P* = 0.70
Having attended a course on malaria management	*P* = 0.80
Having an RDT	*P* = 0.04

## Discussion

The present results show that treatment of pregnant women with fever according to national guidelines was poor in a substantial number of private health facilities, with less than 40 % of providers in private clinics, registered drug shops and pharmacies in rural Uganda knowing the correct first-line treatment for malaria during pregnancy. It is further shown that while majority of providers at private facilities correctly prescribed SP for IPTp, some drug shops also prescribed ACT and quinine for prevention of malaria in pregnancy. These results are compounded by low knowledge that pregnant women are at increased risk of malaria. These results have implications for the safety of pregnant women visiting private health facilities. Of immediate importance for the Ministry of Health is the need to design messages to create awareness that pregnant women are vulnerable to malaria and to distribute the most updated malaria treatment and prevention guideline to private health facilities.

It is shown that less than half of drug shops had rapid diagnostic tests (RDTs) (Table 3). There is potential to scale up use of RDTs in these outlets based on recent findings that showed high adherence to RDT test results and improved appropriate treatment of malaria with ACT in drug shops [17]. Nevertheless, in the private sector where RDTs exist, further operational research will be required to monitor adherence to RDT test results, treatment of non-malaria febrile illnesses and referral practices over time. It is shown that facilities which had RDTs were more likely to treat pregnant women with fever appropriately. This finding justifies the need to scale up this diagnostic tool.

Similar results have been documented in Nigeria where there was little knowledge about treatment and chemoprophylaxis for malaria in pregnancy especially in the private sector; and many providers could not recognize maternal and neonatal deaths as potential consequences of malaria in pregnancy [20]. In Zambia, although not among pregnant women, approximately 30 % of patients who were found negative for malaria parasites were prescribed an anti-malarial drug contrary to the national guidelines [21]. It was further shown that malaria case management was characterized by poor adherence to treatment guidelines, mainly due to inconsistent use of confirmatory tests (rapid diagnostic test or microscopy); prescribing anti-malarial drugs which were not recommended (e.g. sulfadoxine-pyrimethamine) and prescribing anti-malarial drugs to cases testing negative [21].

Although the present results have highlighted deficiencies in treatment practices for fever in pregnancy and the need for improved policy guidance for providers in the private sector; similar concerns might also be raised in the public sector. A recent review showed that no country in sub-Saharan Africa provides appropriate guidance on management of uncomplicated and severe malaria during pregnancy [22]. In all countries, inconsistencies between National Malaria Control programmes and Reproductive Health programmes on the timing or dose of IPTp-SP were documented, as was the mechanism for providing long-term insecticide-treated nets. Inconsistencies were also found in training documents from both programmes. It was concluded that out-dated and inconsistent guidelines had the potential to cause confusion and lead to incorrect practices among health workers who implement malaria in pregnancy programmes, contributing to low coverage of IPTp-SP and long-lasting insecticidal nets (LLINs) [22, 23].

Use and safety of drugs in pregnancy is a major concern. A study has recently shown that trimethoprim is a potential teratogen when used 3 months before pregnancy [24]. It has also been found that Mefloquine as IPTp is not well tolerated, limiting its potential for IPTp and indicating the need to find alternatives with better tolerability to reduce malaria in this particularly vulnerable group [25]. Accordingly, the World Health Organization (WHO) recommends artemisinin-based combination therapy (ACT) to treat uncomplicated falciparum malaria during the second and third trimesters of pregnancy, and quinine plus clindamycin during the first trimester [26]. However, the national policies of many African countries currently recommend quinine throughout pregnancy. Although there are few reports of AL safety in the first trimester additional data are required to assess the potential to use AL in the first trimester [26, 27].

The immediate policy implications of these findings is to distribute the most updated malaria treatment and prevention guideline to private health facilities and to discuss with the Ministry of Health, district leaders, the national drug authority (NDA) and the professional councils that register private providers in Uganda to design continuing medical education (CME) and supervision modalities to ensure quality and safety of patients seeking care at these facilities. It is recommended that further studies to compare the effectiveness and cost of different supervision modalities for the private facilities and how best to link them to the public health system be conducted. Although interventions to improve quality of care in the private sector are recommended, there is caution that pregnant women may miss out on a wider ANC package like HIV testing and access to anti-retroviral therapy (ART) that may exist in the public sector. Thus a clear guideline is required on referral of pregnant women to ANC for other essential maternity care.

A limitation of this study is that treatment practices were never observed when pregnant women visited the facilities; neither was it possible to document what questions women asked when they visited the facilities. This however required an in-depth study that was beyond the scope of this study in terms of time and funds and exploration of these in further research is recommended. Nonetheless, the survey covered a large number of private health facilities and the treatment and prevention practices documented could be generalized to most parts of Uganda.

In conclusion, the study presents disturbing results that show poor treatment and prevention of malaria in pregnancy at private health facilities. Interventions to increase access to effective treatment and prevention of malaria in pregnancy in this sector are urgently needed.

## Abbreviations

ANC: antenatal care
ART: anti-retroviral therapy
RDTs: rapid diagnostic tests
ACT: artemisinin-based combination therapy
IPTp: intermittent preventive treatment
HIV: human immune deficiency virus
NDA: national drug authority
SP: sulfadoxine-pyrimethamine
LLIN: long-lasting insecticidal nets
ITN: insecticide-treated nets

## References

[1] Ministry of Health. Health Sector Development Plan III. Ministry of Health, Box 7272, Kampala; 2015.

[2] Mbonye AK, Bygbjerg IC, Magnussen P (2008). Intermittent preventive treatment of malaria in pregnancy: a new delivery system and its effect on maternal health and pregnancy outcomes in Uganda. Bull World Health Organ.

[3] Ministry of Health. Uganda Clinical Guidelines. Ministry of Health, Box 7272, Kampala, Uganda, 2012.

[4] Uganda Bureau of Statistics (2011). Uganda Demographic and health Survey.

[5] van der Geest S (2006). Self-care and the informal sale of drugs in South Cameroon. Int J Adolesc Med Health.

[6] Mbonye AK, Neema S, Magnussen P (2006). Malaria in pregnancy, risk perceptions and care seeking practices among adolescents in Mukono district Uganda. Int J Adolesc Med Health.

[7] Mbonye AK (2003). Prevalence of childhood illnesses and care-seeking practices in rural Uganda. Scientific World J.

[8] Mbonye AK, Sentongo M, Mukasa GK, Byaruhanga R, Sentumbwe-Mugisa O, Waiswa P (2012). Newborn survival in Uganda: a decade of change and future implications; Uganda Decade of Change and Future Implications Analysis Group. Health Policy Plan.

[9] Ocan M, Bwanga F, Bbosa GS, Bagenda D, Waako P, Ogwal-Okeng J (2014). Patterns and predictors of self-medication in northern Uganda. PLoS ONE.

[10] Heggenhougen HK, Hackenthal V, Vivek P. Bed nets usage and its acceptance at the local level. In: the behaviour and social aspects of malaria control: an introduction and annotated biography. Special program for research and training in tropical Diseases (TDR); 2003:97–106.

[11] Helitzer-Allen DL, McFarland DA, Wirima JJ, Macheso AP (1993). Malaria chemoprophylaxis compliance in pregnant women: a cost-effectiveness analysis of alternate interventions. Soc Sci Med.

[12] Andrews KG, Lynch M, Eckert E, Gutman J (2015). Missed opportunities to deliver intermittent preventive treatment for malaria to pregnant women 2003–2013: a systematic analysis of 58 household surveys in sub-Saharan Africa. Malar J.

[13] Hill J, Hoyt J, van Eijk AM, D’Mello-Guyett L, ter Kuile FO, Steketee R (2013). Factors affecting the delivery, access, and use of interventions to prevent malaria in pregnancy in sub-Saharan Africa: a systematic review and meta-analysis. PLoS Med.

[14] Chico RM, Dellicour S, Roman E, Mangiaterra V, Coleman J, Menendez C, Majeres-Lugand M (2015). Global Call to Action: maximize the public health impact of intermittent preventive treatment of malaria in pregnancy in sub-Saharan Africa. Malar J.

[15] Mbonye AK, Magnussen P, Bygbjerg IB (2007). Intermittent preventive treatment of malaria in pregnancy: the effect of new delivery approaches on access and compliance rates in Uganda. Trop Med Int Health.

[16] Mbonye AK, Bygbjerg IC, Magnussen P (2007). A community-based delivery system of intermittent preventive treatment of malaria in pregnancy and its effect on use of essential maternity care at health units in Uganda. Trans R Soc Trop Med Hyg.

[17] Mbonye AK, Hansen KS, Wamono F, Magnussen P (2010). Integration of malaria and HIV/AIDS prevention services through the private sector in Uganda. Int Health.

[18] Mbonye AK, Lal S, Cundill B, Hansen KS, Clarke S, Magnussen P (2013). Treatment of fevers prior to introducing rapid diagnostic tests for malaria in registered drug shops in Uganda. Malar J.

[19] Mbonye AK, Buregyeya E, Rutebemberwa E, Clarke SE, Lal S, Hansen KS (2016). Prescription for antibiotics at drug shops and strategies to improve quality of care and patient safety: a cross-sectional survey in the private sector in Uganda. BMJ Open.

[20] Onwujekwe OC, Soremekun RO, Uzochukwu B, Shu E, Onwujekwe O (2012). Patterns of case management and chemoprevention for malaria-in-pregnancy by public and private sector health providers in Enugu state, Nigeria. BMC Res Notes.

[21] Chanda-Kapata P, Chanda E, Masaninga F, Habluetzel A, Masiye F, Fall IS (2014). A retrospective evaluation of the quality of malaria case management at twelve health facilities in four districts in Zambia. Asian Pac J Trop Biomed.

[22] Gomez PP, Gutman J, Roman E, Dickerson A, Andre ZH, Youll S (2014). Assessment of the consistency of national-level policies and guidelines for malaria in pregnancy in five African countries. Malar J.

[23] Galactionova K, Tediosi F, de Savigny D, Smith T, Tanner M, Hill J (2015). Insecticide-treated nets. Effective coverage and systems effectiveness for malaria case management in sub-Saharan African countries. PLoS ONE.

[24] Sun Y, Wu CS, Olsen J (2014). Trimethoprim use before pregnancy and risk of congenital malformation: reanalyzed using a case-crossover design and a case-time-control design. Pharmacoepidemiol Drug Saf.

[25] González R, Desai M, Macete E, Ouma P, Kakolwa MA, Abdulla S (2014). Intermittent preventive treatment of malaria in pregnancy with mefloquine in HIV-infected women receiving cotrimoxazole prophylaxis: a multicenter randomized placebo-controlled trial. PLoS Med.

[26] Manyando C, Kayentao K, D’Alessandro U, Okafor HU, Juma E, Hamed K (2012). A systematic review of the safety and efficacy of artemether-lumefantrine against uncomplicated *Plasmodium falciparum* malaria during pregnancy. Malar J.

[27] Manyando C, Njunju EM, Virtanen M, Hamed K, Gomes M, Van Geertruyden JP (2015). Exposure to artemether-lumefantrine (Coartem) in first trimester pregnancy in an observational study in Zambia. Malar J.

